# Recent progress in genetics of aging, senescence and longevity: focusing on cancer-related genes

**DOI:** 10.18632/oncotarget.889

**Published:** 2012-12-30

**Authors:** Albert E. Berman, Olga V. Leontieva, Venkatesh Natarajan, James A. McCubrey, Zoya N. Demidenko, Mikhail A. Nikiforov

**Affiliations:** ^1^ V.N. Orekhovich Institute of Biomedical Chemistry RAMS, 10 Pogodinskaya Str., Moscow, Russia; ^2^ Department Cell Stress Biology, Roswell Park Cancer Institute, Elm and Carlton Streets, Buffalo, NY; ^3^ Department of Microbiology & Immunology, Brody School of Medicine at East Carolina University, Greenville, NC

**Keywords:** senescence, quasi-programmed aging, diseases, cancer

## Abstract

It is widely believed that aging results from the accumulation of molecular damage, including damage of DNA and mitochondria and accumulation of molecular garbage both inside and outside of the cell. Recently, this paradigm is being replaced by the “hyperfunction theory”, which postulates that aging is caused by activation of signal transduction pathways such as TOR (Target of Rapamycin). These pathways consist of different enzymes, mostly kinases, but also phosphatases, deacetylases, GTPases, and some other molecules that cause overactivation of normal cellular functions. Overactivation of these sensory signal transduction pathways can cause cellular senescence, age-related diseases, including cancer, and shorten life span. Here we review some of the numerous very recent publications on the role of signal transduction molecules in aging and age-related diseases. As was emphasized by the author of the “hyperfunction model”, many (or actually all) of them also play roles in cancer. So these “participants” in pro-aging signaling pathways are actually very well acquainted to cancer researchers. A cancer-related journal such as Oncotarget is the perfect place for publication of such experimental studies, reviews and perspectives, as it can bridge the gap between cancer and aging researchers.

## Sirtuins

Sirtuins are NAD+-dependent protein deacetylases, which regulate metabolism, stress responses, and aging processes. Mammalians possess seven Sirtuin isoforms, Sirt1-7, which differ in their subcellular localization and in the substrate proteins they deacetylate [1-6]. Sirtuins remain the most investigated proteins in the field of aging research. In the last several years numerous findings have extended our knowledge on their roles in aging and age-related diseases and cancer [7-22].

Not only there are at least seven unique isoforms with different regulators and substrates, but they can exert opposite effects, including extension and shortening of life span, depending on the isoform, environmental conditions and animal species [1, 14, 23-34]. The biochemical functions of sirtuins are numerous [26, 35, 36]. As one of many examples, SIRT3-mediated deacetylation of cyclophilin D (CypD) on lysine 166 suppressed age-related cardiac hypertrophy [37]. The class III histone deacetylase SIRT1 has been implicated in extension of lifespan. In the vasculature, SIRT1 was demonstrated to improve endothelial function. SIRT1 prevented endothelial superoxide production, inhibited NF-kappaB signaling, and diminished expression of adhesion molecules [38]. Treatment of hypercholesterolemic ApoE−/− SIRT1+/− mice with lipopolysaccharide to boost NF-kappaB signaling, led to a more pronounced endothelial expression of ICAM-1 and VCAM-1. Thus as emphasized by Stein et al, endogenous SIRT1 diminished endothelial activation in ApoE−/− mice [38]. The atheroprotective effects of SIRT1 observed in atherogenesis highlighted the need for additional translational research from bench-to-bedside on this topic [39]. Not coincidentally, modulators of sirtuins are considered as very promising targets for drug development [26, 40-44]. Virtual docking of a compound library into the peptide binding pockets of the crystal structures of Sirt2, 3, 5 and 6 yielded the compounds potentially discriminating between these isoforms. Further characterization in activity assays revealed two compounds with micromolar potency and high specificity for Sirt2 [4]. Sirtuins are also involved in cancer as one example of many age related diseases. For example, SIRT6 overexpression induced massive apoptosis in cancer but not normal cells [45].

## Target of rapamycin

Sirtuins may exert different and sometimes opposite effects on longevity depending on the organism and environmental conditions. It was discussed recently that sirtuins could serve as modulators of the mTOR (mammalian target of rapamycin) pathway, by modulating and predominantly antagonizing mTOR activity, both upstream and downstream [46]. In 2009, Harrison, Miller and co-workers demonstrated that rapamycin, an inhibitor of mTOR, prolonged lifespan in mice [47]. This result was confirmed further by additional independent experiments and in different types (strains) of mice [48-56]. Furthermore, partial genetic inactivation of the mTOR pathway was known to prolong life span in different species from yeast to humans [57-61]. Based on the roles of mTOR in both cellular and organismal aging, as well as in age-related diseases, Mikhail Blagosklonny has predicted that rapamycin might extend lifespan, by slowing aging and delaying age-related diseases [62]. In 2003, he proposed that active growth-promoting pathways that increase cellular size must in fact cause the senescent phenotype, when the cell cycle was blocked and actual cellular growth was not possible [63-64]. Inappropriate activation of some signaling pathways such as mTOR caused cellular hyperfunctions, and contributed to age-related diseases [62]. The theory that aging is a quasi-programmed aimless continuation of developmental growth [62, 65-72], was named the hyperfunction theory by Gems and coworkers, and was experimentally supported by studies in *C elegans* [73, 74].

Recently, the role of mTOR in cellular senescence has been further investigated in a process named geroconversion [75, 76] and was further experimentally supported by studies at the cellular level [77-88]. Besides nutrients, mTOR is also activated by insulin, IGF-1, Ras, PI3K, Raf and other signal transduction molecules [89-93]. All of these signaling molecules are both pro-aging and oncoproteins, making mTOR a central player in both aging and cancer. Rapamycin and other rapalogs such as everolimus and temsirolimus and inhibitors of PI3K (upstream activator of TOR) are being prescribed or undergoing clinical trials for various cancer treatments [94-115].

## Insulin and IGF

Reduced insulin and IGF-1 signaling has been associated with animal and human longevity [116-121].On the other hand, inhibition of insulin/IGF-1 signaling is one anti-cancer strategy under intensive investigation [122-129].

## Ras and PI3K

Ras and PI3K are potent inducers of cellular senescence, especially when cells cannot respond by increased proliferation [130-138]. Ras also participates in activities related to aging such as increased metabolism and autophagy [139]. The link between Ras and lifespan was further elucidated in a RasGrf1-deficient mouse model [140]. RasGRF1 is a Ras-guanine nucleotide exchange factor implicated in a variety of physiological processes. In aged RasGrf1(−/−) mice, increases in average and maximal lifespan, were associated with lower IGF-I levels and increased SIRT1 levels. Life extension was not due to the role of Ras in cancer or a protection against oxidative stress. In addition, cardiac glucose consumption was changed by aging in the mutant mouse model, indicating that RasGrf1-deficient mice displayed elevated aging [140-142]. Additional work supporting the role of Ras in organismal aging, demonstrated that Ras can accelerate aging [118, 143, 144], consistent with ‘the hyperfunctional model’ of aging driven by growth-promoting activators of the mTOR global network. Needless to say, Ras, Raf, PI3K and Akt are some of most important players in cancer and therefore targets for therapy. Recently clear progress in therapeutic applications of inhibiting these targets has been demonstrated [98, 104, 107,112, [144-155]

## p53

The p53 tumor suppressor is one of the most famous inducers of cellular senescence [134, 137, 156-161]. Moreover, this outcome was demonstrated to be ensured when p53 caused cell cycle arrest but failed to inhibit the mTOR pathway [162]. By inhibiting mTOR [163], p53 can suppress the senescence program, the senescent phenotype and associated morphology, resulting in reversible arrest [164]. Since p53 can inhibit mTOR under certain conditions in various cells, it may cause quiescence instead of senescence [165-171]. p53 was demonstrated to inhibit geroconversion (a conversion from quiescence or simple arrest to senescence [76]) and, importantly, it did not cause senescence in quiescent cells [79]. Not surprisingly, the effects of p53 on longevity may vary [33, 172-180]. On the other hand, since p53 is the most frequently mutated tumor suppressor gene, p53 is under further investigations for various cancer therapies to characterize and develop new drugs and approaches for targeting both mutated and WT p53 [181-194].

HIF-1 is often induced in cancer in response to hypoxic conditions, which by the way inhibit senescence in a HIF-1-independent fashion. Interestingly, HIF-1alpha protects against drug-induced apoptosis by antagonizing the functions of p53 [195]. HIF-1alpha upregulation induced proteasomal degradation of homeodomain-interacting protein kinase-2 (HIPK2), a p53 apoptotic activator [195]. Agents that target HIF-1 are under further development [196-202].

Another strategy is induction of p53 for protection of normal cells from cycle-dependent chemotherapy, currently known as chemo-cyclo-therapy or cyclo-therapy [203-209].

## p63 and p73

p63 and p73, relatives of p53, play even more diverse role in aging [210-215]. One unusual pro-aging role of p73 has been recently demonstrated. Female reproductive aging is often associated with increases in egg aneuploidy [216]. It was observed that TAp73 isoforms were down regulated in oocytes from women older than 38 years. TAp73 down regulation in oocytes from women of advanced reproductive age could explain both the reduction of fertility and the increase in frequency of newborns with chromosomal abnormalities [216]. p63 and p73 are also important targets for anti-cancer therapies [204, 217-230].
